# The effect of simulation of sectional human anatomy using ultrasound on students’ learning outcomes and satisfaction in echocardiography education: a pilot randomized controlled trial

**DOI:** 10.1186/s12909-024-05337-x

**Published:** 2024-05-03

**Authors:** Kewen Ding, Mingjing Chen, Ping Li, Zichuan Xie, Haorong Zhang, Ruixing Kou, Jionghui Xu, Ting Zou, Zhen Luo, Haibo Song

**Affiliations:** 1 West China School of Medicine, Sichuan University, 610041 Chengdu, Sichuan P. R. China; 2 Department of Epidemiology, College of Public Health & Health Professions and College of Medicine, University of Florida, Gainesville, FL USA; 3 Department of Anesthesiology, West China Hospital, Sichuan University, 610041 Chengdu, Sichuan China

**Keywords:** Echocardiography Teaching, Simulation, Sectional human anatomy, Learning outcome

## Abstract

**Background:**

Effective teaching methods are needed to improve students’ abilities in hand-eye coordination and understanding of cardiac anatomy in echocardiography education. Simulation devices have emerged as innovative teaching tools and exhibited distinctive advantages due to their ability to provide vivid and visual learning experiences. This study aimed to investigate the effect of simulation of sectional human anatomy using ultrasound on students’ learning outcomes and satisfaction in echocardiography education.

**Methods:**

The study included 18 first-year clinical medical students with no prior echocardiography training. After randomization, they underwent a pre-test to assess basic knowledge. Following this, the students were divided into two groups: traditional teaching (traditional group) and simulation of sectional human anatomy using ultrasound (digital group). Each group received 60 min of instruction. Post-tests were assigned to students at two different time points: immediately after the lecture, and one week later (referred to as post-tests 1, and 2). In addition, anonymous questionnaires were distributed to students after class to investigate their satisfaction with teaching.

**Results:**

Both groups showed significant improvement in their scores on post-test 1 compared to pre-test (traditional group: from 33.1 ± 8.8 to 48.1 ± 13.1, *P* = 0.034 vs. digital group: from 35.0 ± 6.7 to 58.0 ± 13.2, *P* = 0.008). However, there were no significant differences between the two groups in several post-test comparisons. Student satisfaction ratings revealed that the digital group experienced significantly greater satisfaction in areas such as subject interest, teaching style, course alignment, and interaction compared to the traditional group. Additionally, 80% of the digital group strongly endorsed the use of simulation of sectional human anatomy using ultrasound for echocardiography teaching, highlighting its effectiveness.

**Conclusions:**

Simulation of sectional human anatomy using ultrasound may improve students’ understanding of echocardiography and satisfaction with the course. Our study provides evidence supporting the use of simulation teaching devices in medical education. Further research is needed to explore the long-term impact of this teaching method on students’ learning outcomes and its integration into the medical curriculum.

**Trial registration:**

http://www.chictr.org.cn (registration number: ChiCTR2300074015, 27/07/2023).

**Supplementary Information:**

The online version contains supplementary material available at 10.1186/s12909-024-05337-x.

## Background

The use of point-of-care ultrasound for cardiac indications has been widely embraced by medical workers, especially emergency medicine providers, due to its advantages such as portability, speed, safety, and low cost [1, 2]. Notably, research indicates that non-cardiologist intensivists, with minimal formal training, have successfully executed and accurately interpreted transthoracic echocardiography (TTE) in critical care settings [3]. Another study discussed the feasibility of integrating an ultrasound-based course into the conventional undergraduate medical teaching program, and reaching positive conclusions [4]. 

In recent years, echocardiography education has undergone a transformation, transitioning from traditional textbooks featuring static images to dynamic multimedia education and interactive websites, enhancing learning through interactivity and diverse media support. For example, a study has shown that a routine 10-hour online echocardiographic course can lead to significant improvement in the ability of 5th-year medical students to interpret cardiac echo data [5]. Another research has shown that a combined video-based student-tutor approach can result in similar knowledge acquisition as compared to a faculty staff-led course without media support [6]. Additionally, teaching software has also proven to be an effective means of instruction [7]. Researchers have developed software for browsing various sections of a heart model to help medical students become familiar with the clinical images of the heart [8], and a study has implemented this process on smartphones, providing convenience for learning [9]. For a narrower set of skills, software can also help students efficiently master knowledge through seeing a high volume of exams, namely perceptual learning modules (PLMs) [10]. 

Despite these advancements, echocardiography education still confronts challenges in effectively bridging the gap between theoretical knowledge and practical skills. Many students struggle with understanding fundamental ultrasound physics, cardiac anatomy, blood flow hemodynamics, ultrasound manipulation techniques, and the interpretation of ultrasound images, particularly in understanding the precise positioning and manipulation of the probe to obtain accurate cardiac sections. For example, an interview with third-year medical students showed that for beginners in echocardiography, it was particularly difficult to know where the probe should be placed, angled and turned to obtain certain sections. [11]. It would be very helpful if pedagogical equipment could connect the description of orientation in books with the placement of the probe, thereby bridging the theory-practice gap. Existing literature [12, 13] often focuses on the outcomes of traditional or digital learning methods separately, but there is a lack of comprehensive research that compares these approaches in the context of hands-on, practical skills in echocardiography.

Simulation-based training, using high-fidelity simulation devices, is an emerging teaching tool that can simulate clinical scenarios to help students practice operations. Numerous studies have confirmed the effectiveness of high-fidelity simulation in teaching echocardiography, as it enhances self-confidence, knowledge and skills, and improves organizational practice among a variety of learners in different clinical settings [14, 15]. For example, some studies found no significant differences between echocardiography training on human models and high-fidelity simulation for undergraduate medical students [16, 17]. Another study reported that simulation-based training, compared to video-based training, significantly improved the performance of medical students following a 90-minute theoretical lecture [18]. 

Traditionally, the predominant pedagogical approach has been the theoretical lecture, a method that principally employs texts and images to convey knowledge. This method tends to compartmentalize the educational process into discrete stages: comprehension, memorization, and application. Such a division can render the learning experience somewhat laborious and uninspiring for students. Although this approach holds intrinsic merit in terms of foundational effectiveness, it presents substantial opportunities for enhancement to better engage and educate learners. Effective teaching methods are crucial for improving students’ abilities in echocardiography education. This study aims to compare the traditional teaching method with the use of simulation of sectional human anatomy using ultrasound (digital human; refer to the Methods section for details) and examine the differences in students’ performance and satisfaction levels between the two approaches.

## Methods

### Study design

We conducted a single-center, randomized, controlled, prospective clinical trial at the West China School of Medicine, Sichuan University from November 2022 to December 2022. The trial was registered with the Chinese registry of clinical trials at http://www.chictr.org.cn (registration number: ChiCTR2300074015, 27/07/2023). The research protocol was approved by the Biomedical Ethics Committee of West China Hospital, Sichuan University.

### Participants

The recruiting time was from November 18, 2022 to December 31, 2022. A total of 18 first-year medical students from Sichuan University’s College of Medicine were recruited for the study. These students, from departments of Clinical Laboratory Sciences (10 students), Clinical Medicine (5 students), and Nursing Science (3 students), had no prior echocardiography training. This diverse cohort ensured a uniform baseline of knowledge and skills. All participants provided informed written consent, complying with ethical standards and understanding the study’s objectives and procedures.

The study’s capacity was limited due to the small size of the seminar room and the challenges in handling the teaching equipment, necessitating a maximum of 10 students per session. Additionally, the COVID-19 pandemic further constrained our ability to conduct multiple teaching sessions, limiting our total participant count to 20. This cap was essential for maintaining high-quality instruction and adhering to safety protocols during the pandemic. We believe that smaller class sizes facilitate more effective interaction, personalized attention, and a better overall educational experience. This deliberate limitation in participant numbers ensures a more focused and engaging learning environment.

### Randomization

The participants were randomly into the traditional and digital groups using sealed envelopes generated by SPSS with random seed 20,221,118.

### Intervention

**Fig. 1:**
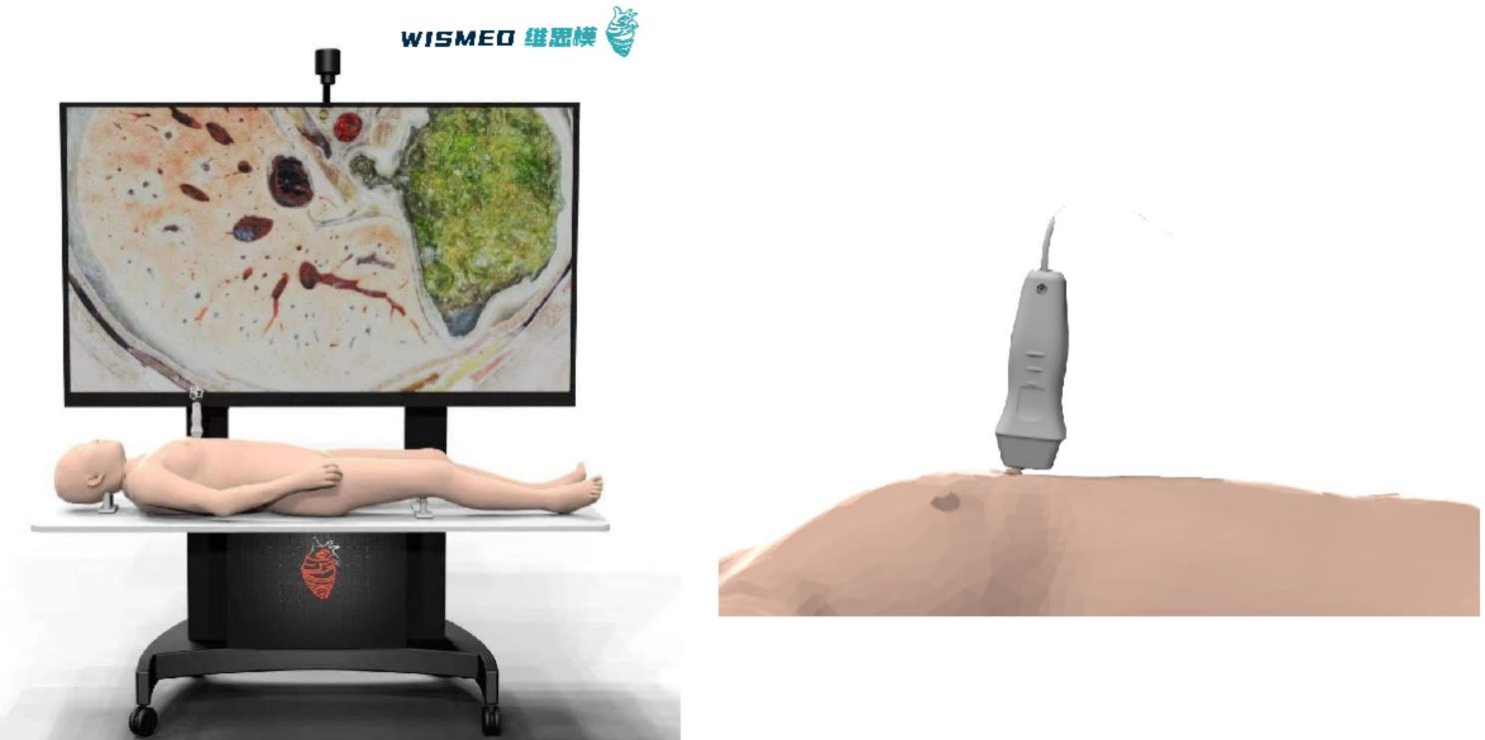
Simulation of sectional human anatomy using ultrasound (also known as “digital human”)

**Fig. 2:**
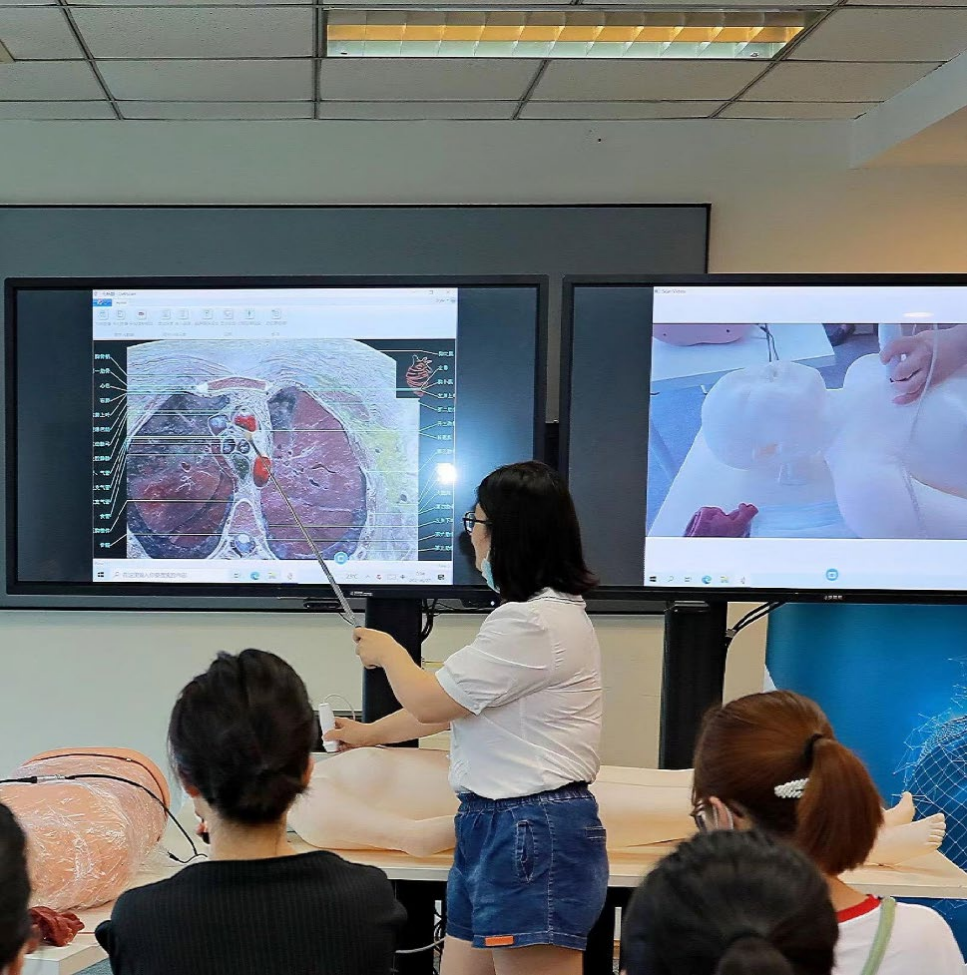
Realistic application of the digital human

The participants in the traditional group received a 60-min theoretical lecture, while the digital group was shown the cross-sectional views through the digital human model (Fig. 1) for 60 min by the teacher. Figure 2 shows the realistic application of the digital human. In the 60-min training sessions, both groups received identical teaching content from the same teacher: the basic concepts of echocardiography, normal cardiac anatomy, a simple explanation of 4 basic cross-sectional views, and the corresponding anatomical structures.

The two experienced teachers involved in this study have been specialized in the field of echo-training for over 9 years, each accumulating over 100 teaching hours annually, and possess extensive teaching experience.

### Simulation of sectional human anatomy using ultrasound

Simulation of sectional human anatomy using ultrasound (also known as “digital human”) used in this study is a unique and advantageous teaching tool among various simulation devices. The digital human adopts Chinese visible human data, which is collected from complete disease-free corpses [19, 20]. A total of 3760 pieces of data were included (head and neck slices with an interval of 0.25 mm, and the rest of the slices with an interval of 0.5 mm). The resolution of a single piece is as high as 3072*2048 pixels, and the arterial perfusion was performed, with clear and distinguishable blood vessels, which is more realistic, intuitive, and three-dimensional compared to traditional teaching methods. In terms of computation, the imaging time of a single slice is less than 0.03 s, achieving real-time display function. The accurate positioning of the probe during echocardiography allows users to directly visualize cardiac anatomy in a manner that corresponds to its actual visual representation. With the aid of digital human, it facilitates the creation of highly precise 3D reconstructions of cardiac structures, resulting in an exceptional level of accuracy when depicting real anatomy. Furthermore, this interactive environment allows for the dissection and exploration of these anatomical structures, providing users with a comprehensive and immersive learning experience. This operating method allows students to view slice images from any position and angle, further enhancing their understanding of the internal structure of the human body. At the same time, the digital human provides detailed annotations to meet the needs of students’ learning in anatomy, ultrasound imaging, and related fields. A previous study using a similar device for transesophageal echocardiography (TEE) teaching showed that digital human has advantages in helping students interpret ultrasound images due to their intuitive, vivid, and interactive features [18]. In summary, the digital human can provide a more vivid and visual teaching method, stimulate students’ learning interests, improve teaching effectiveness, and provide better conditions for the cultivation of clinical doctors.

### Measurements

After the randomization, we conducted a pre-test to evaluate students’ basic knowledge. Following the completion of the intervention measures for both groups, two post-tests were conducted to evaluate the teaching effect and students’ memory: the post-test 1 was conducted immediately after the intervention, the post-test 2 was conducted 1 week after the intervention.

The testing content of the study mainly included four basic sections of TTE: left ventricular long axis section, left ventricular papillary muscle short axis section, right ventricular inflow and outflow tract section, and four chamber section. These four basic sections correspond to four cardiac-focused sections out of the six basic sections of TEE, with the two unselected sections pertaining to the great vessels [21]. For each test, 20 questions were randomly selected from a question bank for each student. Each question has 4 options, and the time for each test was limited to 15 min. We used the accuracy (%) of each student to evaluate their performance in each test. The composition of the question bank is shown in Supplementary Material 1.

In addition, after the intervention, we distributed an anonymous questionnaire to students. This study used Student Evaluation of Educational Quality Questionnaire (SEEQ) [22] to evaluate the effectiveness of using a digital human model in a course. To make the questionnaire more relevant to the study, we removed some inapplicable items such as individual rapport, breadth of coverage, examinations/grading, assignments, and part of workload/difficulty, which were irrelevant to this study and unanswered by the students, and added a new question based on the content of our research: whether the students accepted the experience of using the digital human model (only the digital group was required to answer). The scores for each item were divided into five grades, ranging from 1 to 5 (strongly disagree, disagree, neutral, agree, strongly agree). The only exception was the question related to the pace of the course, which was rated on a scale from 1 (too slow) to 5 (too fast).

### Statistical analysis

All data were presented as mean ± standard deviation (SD). We used Mann-Whitney U test to test the difference of performances. Alpha was set at 0.05, and P-values of less than 0.05 were considered significant. Data for this study were collected using Microsoft Excel and analyzed using IBM SPSS version 27.0.1.0 (IBM Corporation, Armonk, New York).

### Students’ performance assessment

The primary outcome was the comparison of the performances (%) in the post-test 1 between the two groups. The secondary outcomes included: (1) the comparison of the performances in the pre-test, and post-test 2 between the two groups; (2) the comparison of the performances in each group between the pre-test and post-test 1; (3) the comparison of the scores in each item of the questionnaire between the two groups.

## Results

### Study population

The basic information of all the participants is presented in Table 1. There was no significant difference between the two groups.

**Table 1:** Comparison of characteristics between traditional group and digital group

Item		Traditional group (*n* = 8)	Digital group (*n* = 10)	P value
Age		18.25 ± 0.46	18.30 ± 0.48	0.819
Sex	Male (%)	25	30	1.000
	Female (%)	75	70	
Grade		Junior college students		
Any previous echocardiography training experience?		No		

**Fig. 3:**
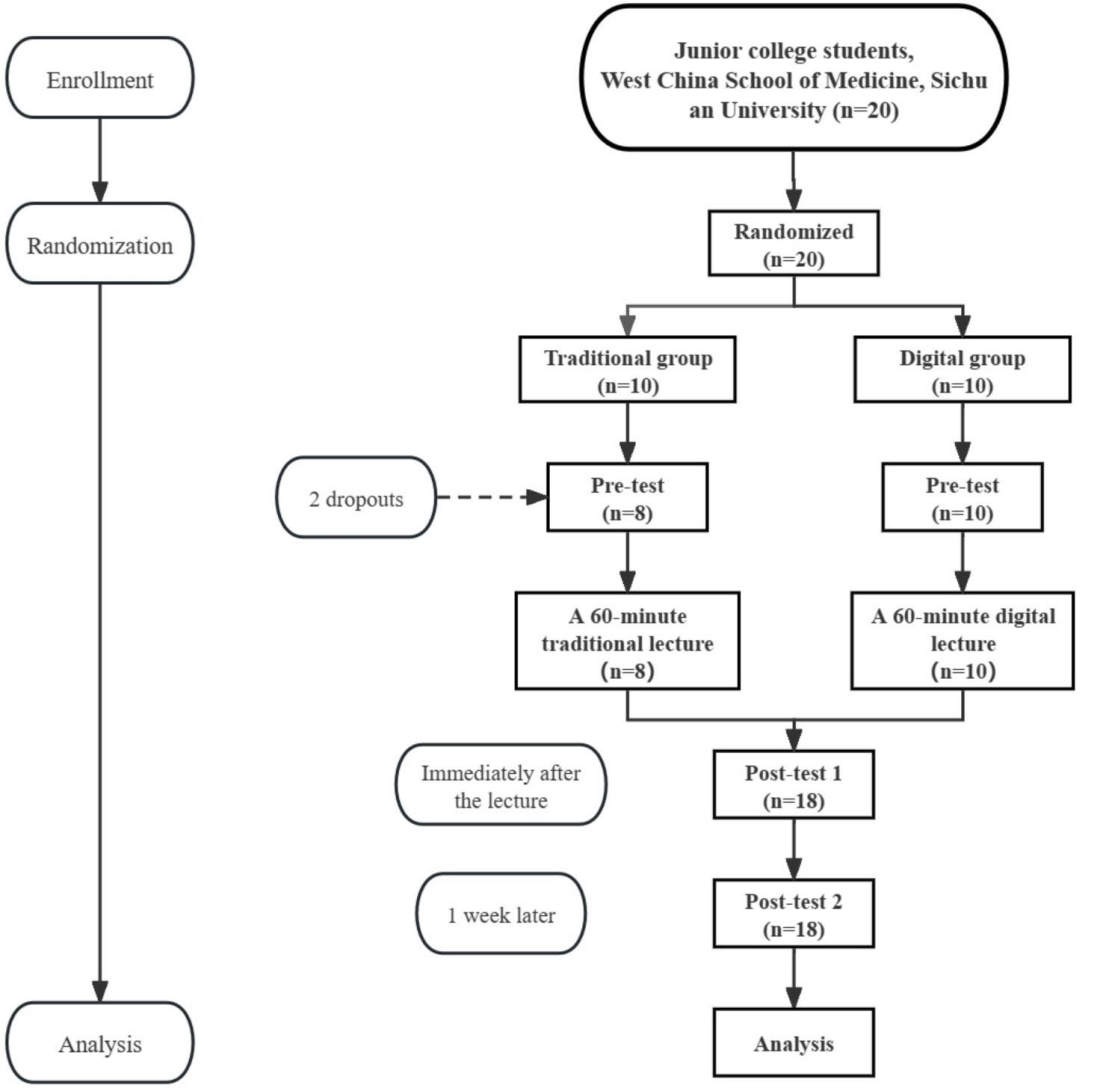
Flow chart of the echocardiography training sessions and tests. *Note*: Dropouts refer to students who voluntarily withdrew from the study

Figure 3 illustrates the sequential progression of echocardiography training sessions and tests. Following the recruitment phase, 20 students willingly participated in the study. However, it’s important to note that two students from the traditional group withdrew during the course of the study.

### Students’ performance analysis

The comparison of the performances (%) in each test between the traditional group and the digital group is shown in Table 2. There was no significant difference in the pre-test between the two groups. In the post-test 1, the digital group performed better in terms of the position and orientation of the probe (hereinafter referred to as “section position”), but no significant difference was shown (traditional group: 31.7 ± 22.3 vs. digital group: 54.4 ± 19.3, *P* = 0.051).

**Table 2:** The comparison of the performances (%) in each test between the traditional group and the digital group

Item		Traditional group (*n* = 8)	Digital group (*n* = 10)	Statistics	P value
Pre-test	Total	33.1 ± 8.8	35.0 ± 6.7	U = 76.00	1.000
	Section content	33.4 ± 16.5	34.5 ± 4.2	U = 75.00	0.929
	Section position	35.0 ± 29.3	38.0 ± 28.3	U = 75.50	0.964
	Section function	29.2 ± 26.4	50.0 ± 36.3	U = 59.50	0.216
	Other types	25.0 ± 46.3	18.3 ± 24.2	U = 74.00	0.832
Post-test 1	Total	48.1 ± 13.1	58.0 ± 13.2	U = 61.50	0.191
	Section content	53.4 ± 14.5	58.9 ± 15.0	U = 68.00	0.477
	Section position	31.7 ± 22.3	54.4 ± 19.3	U = 54.50	0.051
	Section function	33.3 ± 51.6	37.8 ± 38.1	U = 45.00	0.493
	Other types	28.1 ± 27.1	25.0 ± 24.8	U = 79.50	0.747
Post-test 2	Total	40.6 ± 20.8	48.5 ± 12.7	U = 67.50	0.446
	Section content	43.8 ± 30.4	50.1 ± 16.3	U = 69.50	0.563
	Section position	29.8 ± 26.9	57.4 ± 29.7	U = 54.50	0.055
	Section function	48.8 ± 38.0	60.0 ± 37.0	U = 55.00	0.425
	Other types	45.8 ± 42.5	23.3 ± 26.3	U = 89.00	0.223

*Note*: 1. The number of questions completed by each student was not given any weight

2. Questions that students fail to answer due to overtime are only included in the statistics of the total performance, and are not included in the statistics of the performance of various types of questions

Table 3 presents the comparison of the performances (%) in each group between the pre-test and the post-test 1. There were significant differences in the performances of the two groups (traditional group: from 33.1 ± 8.8 to 48.1 ± 13.1, with an absolute gain of 15.0 and a relative gain of 45.32%, *P* = 0.034 vs. digital group: from 35.0 ± 6.7 to 58.0 ± 13.2, with an absolute gain of 23.0 and a relative gain of 65.71%, *P* = 0.008), mainly in terms of section content (traditional group: from 33.4 ± 16.5 to 53.4 ± 14.5, with an absolute gain of 20.0 and a relative gain of 59.88%, *P* = 0.042 vs. digital group: from 34.5 ± 4.2 to 58.9 ± 15.0, with an absolute gain of 24.4 and a relative gain of 70.72%, *P* = 0.008). As previously defined, “absolute gain” (%) refers to the difference between the mean performances in the post-test 1 and the pre-test, and “relative gain” refers to the ratio of absolute gain to the mean performance in the pre-test [23]. 

**Table 3:** The comparison of the performances (%) in each group between the pre-test and the post-test 1

Item		Statistics	P value
	**Total** ^*^	U = 1.50	0.034
	**Section content** ^*^	U = 2.00	0.042
Traditional group (*n* = 8)	Section position	U = 16.00	0.779
	Section function	U = 3.00	0.458
	Other types	U = 10.00	0.916
	**Total** ^**^	U = 0.00	0.008
	**Section content** ^**^	U = 0.00	0.008
Digital group (*n* = 10)	Section position	U = 9.50	0.233
	Section function	U = 4.50	0.196
	Other types	U = 10.00	0.496

*Note*: 1. Specific data are shown in Table 2

2. ^*^represents *P* < 0.05, ^**^represents *P* < 0.01

**Fig. 4:**
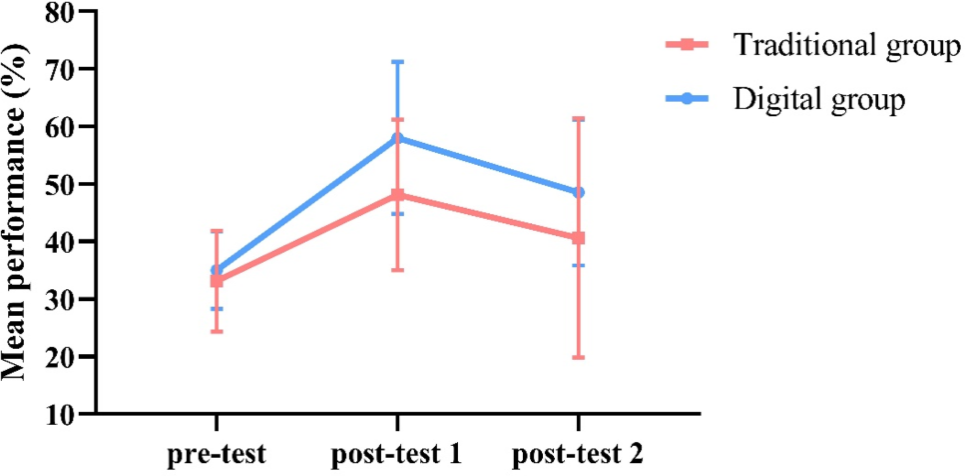
Changes in student performances (total)

Figure 4 presents changes in student performances (total) of the two groups.

### Questionnaire result analysis

The specific content of each item in the questionnaire is shown in Table 4, and Table 5 presents the comparison of the scores and the percentage of students who strongly agreed in each item between the two groups. To evaluate the questionnaire’s reliability, we calculated the Cronbach’s alpha coefficient, which was 0.755 (indicating good reliability) after excluding the question related to course pace, indicated that the modified SEEQ was a reliable instrument.

**Table 4:** The specific content of each item in the questionnaire

Item	Question
Learning value	
Q1	I have found the course intellectually challenging and stimulating.
Q2	I have learned something which I consider valuable.
Q3	My interest in the subject has increased as a consequence of this course.
Q4	I have learned and understood the subject materials of this course.
Overall evaluation	
Q5	Compared with other courses I have had, I would say this course is:
Q6	Compared with other instructors I have had, I would say this instructor is:
Q7	As an overall rating, I would say this instructor is:
Enthusiasm of the teacher	
Q8	Instructor was enthusiastic about teaching the course.
Q9	Instructor was dynamic and energetic in conducting the course.
Q10	Instructor enhanced presentations with the use of humor.
Q11	Instructor’s style of presentation held my interest during class.
Organization of teaching	
Q12	Instructor’s explanations were clear.
Q13	Course materials were well prepared and carefully explained.
Q14	Proposed objectives agreed with those actually taught so I knew where course was going.
Q15	Instructor gave lectures that facilitated taking notes.
Group interaction	
Q16	Students were encouraged to participate in class discussions.
Q17	Students were invited to share their ideas and knowledge.
Q18	Students were encouraged to ask questions and were given meaningful answers.
Q19	Students were encouraged to express their own ideas and/or question the instructor.
Workload/Difficulty	
Q20	Course pace (too slow-too fast)
Other aspect	
Q21	Acceptance of the experience of using digital human model in the course (only for the digital group)

*Note*: Q means question

**Table 5:** The comparison of the questionnaire results between the traditional group and the digital group

	Traditional group (*n* = 8)		Digital group (*n* = 10)			
Item	Mean ± SD	Strongly agree (%)	Mean ± SD	Strongly agree (%)	Statistics	P value
Q1	4.63 ± 0.52	62.5	4.50 ± 0.53	50.0	U = 81.00	0.606
Q2	4.50 ± 0.76	62.5	4.60 ± 1.27	90.0	U = 66.50	0.246
**Q3** ^**^	3.88 ± 0.64	12.5	4.80 ± 0.42	80.0	U = 47.00	0.004
Q4	3.50 ± 0.93	12.5	4.00 ± 0.82	20.0	U = 62.00	0.170
**Q5** ^*^	4.00 ± 0.54	12.5	4.60 ± 0.52	60.0	U = 55.00	0.034
Q6	4.00 ± 0.76	25.0	4.60 ± 0.52	60.0	U = 58.00	0.078
**Q7** ^*^	4.13 ± 0.64	25.0	4.80 ± 0.42	80.0	U = 53.00	0.020
**Q8** ^*^	4.50 ± 0.76	62.5	5.00 ± 0.00	100.0	U = 61.00	0.040
**Q9** ^*^	4.50 ± 0.54	50.0	5.00 ± 0.00	100.0	U = 56.00	0.014
Q10	3.88 ± 0.84	25.0	4.30 ± 0.48	30.0	U = 63.50	0.216
**Q11** ^**^	3.50 ± 0.54	0.0	4.70 ± 0.48	70.0	U = 42.00	0.001
**Q12** ^*^	4.25 ± 0.71	37.5	4.90 ± 0.32	90.0	U = 54.50	0.021
**Q13** ^*^	4.13 ± 0.84	37.5	4.90 ± 0.32	90.0	U = 54.00	0.019
**Q14** ^**^	3.75 ± 0.71	12.5	4.70 ± 0.48	70.0	U = 48.50	0.008
**Q15** ^*^	3.63 ± 1.06	25.0	4.70 ± 0.48	70.0	U = 52.00	0.021
**Q16** ^*^	3.63 ± 0.74	12.5	4.50 ± 0.71	60.0	U = 52.50	0.026
**Q17** ^***^	3.38 ± 0.52	0.0	4.80 ± 0.42	80.0	U = 39.00	< 0.001
**Q18** ^***^	3.63 ± 0.52	0.0	4.90 ± 0.32	90.0	U = 38.50	< 0.001
**Q19** ^*^	3.75 ± 0.71	12.5	4.60 ± 0.52	60.0	U = 51.00	0.016
Q20	3.50 ± 0.54		3.50 ± 0.53		U = 76.00	1.000
Q21			4.80 ± 0.42	80.0		

*Note*: 1. ^*^represents *P* < 0.05, ^**^represents *P* < 0.01, ^***^represents *P* < 0.001

2. The specific content of each item in the questionnaire is shown in Table 4

Overall, the feedback of the digital group was better than that of the traditional group in learning value, overall evaluation, enthusiasm of the teacher, organization of teaching, and group interaction, with significant differences especially in the last two aspects. There were significant differences in scores of all the questions. The difference between the two groups was particularly significant in the following five aspects of feedback (*P* < 0.01): The students in the digital group reported a higher agreement with statements such as “my interest in the subject has increased as a consequence of this course,” “instructor’s style of presentation held my interest during class,” “proposed objectives agreed with those actually taught so I knew where course was going,” “students were invited to share their ideas and knowledge,” and “students were encouraged to ask questions and were given meaningful answers.”

In addition, when asked about acceptance of the experience of using digital human model in the course (the question mentioned above that requires only the digital group to answer), the students gave high scores (4.80 ± 0.42), with 80.0% of them strongly agreeing.

## Discussion

The findings from our study indicated that, in terms of basic echocardiography teaching, students who received digital human teaching did not exhibit lower overall test scores compared to those who received traditional teaching. Additionally, students in the digital teaching group expressed higher satisfaction with the teaching approach.

The scores of both groups in the post-test 1 were significantly higher than those in the pre-test, which indicates that both teaching methods have certain effects. The fact that there was no significant difference in the pre-test proves that the two groups of students had the same level of basic knowledge before receiving teaching. On this basis, we observed no significant difference in the two post-test scores between the two groups, which is consistent with the results of some previous studies on simulation-based teaching [24, 25]. This indicates the basic effectiveness of digital human as a simulation device, but on the other hand, in terms of data, although not showing significance, the performances of the digital group were to some extent superior than that of the traditional group, and it could potentially exhibit statistical differences in larger sample sizes. At the same time, we have also observed that after undergoing different trainings, the digital group showed better performance in terms of section position in post-test 1 compared to the traditional group (although no significant difference was shown due to the small sample size), which is consistent with our previous assumptions; however, in other aspects (excluding the section position), the learning effect of the traditional group was higher than expected (with no significant difference compared to the digital group), possibly due to the low level of understanding required for mastering these knowledge, and the students’ learning focus during the experiment was higher than that of regular classes, resulting in better memory effects. Anyway, the qualified performance of the digital group in section position suggests that the use of the digital human may provide a more effective means of teaching echocardiography to medical students. This may be attributed to the fact that the digital human allows students to view anatomical structures in a more realistic and interactive manner, as well as the ability to rotate and manipulate the image to visualize it from different angles. This may enhance students’ understanding of the spatial relationships between anatomical structures and facilitate the learning of echocardiography. After all, ultrasound simulation equipment, as a real-time operating device, requires a high level of anatomical knowledge from users, and it is also an effective tool for learning anatomy. Research has shown that the ultrasound simulator appears equivalent to human cadaveric prosections for learning cardiac anatomy [26]. On the other hand, the digital human can provide immediate and effective feedback on students’ operations, which is a significant help for echocardiography learning [27], especially in the “section position” where theory and practice need to be combined.

Furthermore, our findings suggest that the use of the digital human may enhance students’ satisfaction with the course, particularly in the areas of teaching organization and group interaction. The students in the digital group also expressed a high acceptance of using the digital human for teaching. This may be due to the increased interactivity and engagement that the digital human provides, as well as the ability to facilitate group discussion and collaborative learning. Recent evidence suggests that millennial medical students value curriculum flexibility and hope to autonomously control the pace of their learning experience [28]. Therefore, digital human simulation teaching that provides opportunities for autonomous operation is more in line with the learning style of modern students, which may be the reason for the high satisfaction feedback from participants in the digital group. Our findings are consistent with a previous study that has reported the advantages of simulation-based teaching methods in enhancing students’ satisfaction and engagement [29]. Specifically, apart from the evaluation of the lecture itself, students in the digital group also generally expressed that “my interest in the subject has increased as a consequence of this course,” which may have important potential benefits for long-term teaching and students’ autonomous learning. Interest can serve to heighten involvement, concentration, attention, intrinsic motivation, as well as positive emotions, all of which foster deep understanding of a given subject area [30]. In the field of echocardiography teaching, further research is needed to transform interest into practical results.

The teaching experiment conducted in this study focuses on the basic knowledge of echocardiography. At present, the digital human remains a tool for learning a normal TTE examination only and lacks one of the distinctive features of a simulator, which cannot simulate a host of pathologic conditions [31]. In the current situation that good results have been achieved, we can expect the digital human to also play a role in high-level echocardiography teaching, including disease diagnosis and other aspects. In larger scale and longer class teaching, there is also the possibility of using the digital human to integrate basic knowledge and advanced clinical knowledge, thereby constructing a systematic, complete, and logical teaching system. In addition, further research is needed to explore the potential benefits and limitations of using the digital human in teaching other areas of medical education. For example, existing literatures have pointed out that simulation devices also have broad application prospects in the teaching of TEE [31–33], neonatal echocardiography [34], workflow in echocardiography [35] and other fields.

Several limitations of our study should be noted. First, the sample size, constrained by seminar room space and the COVID-19 pandemic, was relatively small, potentially limiting the statistical power and breadth of our data collection. These constraints were crucial for effective space utilization and adherence to health guidelines but did limit the scope of our research. Moreover, with the withdrawal of two participants, there was a slight deviation between the actual and the calculated theoretical sample size (Fig. 3). This discrepancy might have contributed to the lack of significant differences in post-test scores between the traditional and digital groups. However, it is important to note that our study was designed as a pilot, characterized by smaller sample sizes for feasibility testing and preliminary data collection in a controlled setting, allowing for detailed feedback and close monitoring of the teaching process. Second, the cost of digital humans is relatively high, and for some educationally resource-limited educational settings, digital humans may not be a practical and feasible teaching tool. It is worth noting that while the digital human provides a more engaging and interactive means of teaching echocardiography, it is not intended to replace traditional teaching methods entirely. Rather, it should be used as a complementary tool to enhance the learning experience of medical students. Third, we used a standardized questionnaire to evaluate students’ satisfaction and did not collect free-text comments, which could have provided deeper insights into student preferences for the “digital human” method. This, along with a feedback mechanism that still requires optimization, will be addressed in our upcoming randomized controlled trial by including a free-text comment section. Furthermore, potential misclassification bias arose from dividing test items into only four categories. Future studies might consider further subdividing these categories for more precise results. Additionally, the small total number of questions in our question bank may explain the lack of significant differences in various question types and overall scores. Last, our study focused on the short-term benefits of simulation teaching, not addressing the longer-term memory retention of students, which is an area for future research.

However, our study has several strengths. To date, few studies have verified the application of similar digital human models in the teaching of echocardiography. Compared with previous simulation teaching methods, digital human simulation teaching has certain advantages (as explained earlier), and these advantages are more evident in the observation of ventricles and valves when learning echocardiography. In terms of performance testing, we classified the test questions based on their content to observe the impact of digital human teaching on different knowledge points. We also conducted multiple tests at various time points to observe the memory effect. In addition, we released an anonymous questionnaire after class to collect subjective feedback from students, thereby overcoming the shortcoming of many previous studies that replied solely on objective grades to evaluate learning outcomes.

## Conclusion

Our study suggests that the use of the digital human may provide a more effective and engaging means of teaching echocardiography to medical students. The use of such technology may enhance students’ understanding of echocardiography and their overall satisfaction with the course. These findings contribute to the growing evidence supporting the use of simulation teaching devices, such as the digital human, in medical education. The integration of digital human models in echocardiography education has the potential to enhance students’ learning experiences and improve their competency in this important field.

Future research should focus on investigating the long-term impact of this teaching method on students’ learning outcomes, including retention and transfer of knowledge. Additionally, further studies are needed to explore the feasibility of incorporating simulation teaching devices into the medical curriculum and to compare different types of simulation devices and to integrate multiple teaching modalities.

### Electronic supplementary material

Below is the link to the electronic supplementary material.

Supplementary Material 1

## Data Availability

The datasets generated during the current study are available in the ResMan Clinical Trial Management Public Platform, http://www.medresman.org.cn/pub/cn/proj/projectshshow.aspx?%20proj=5295http://www.medresman.org.cn/pub/cn/proj/projectshshow.aspx?proj=5295.

## Abbreviations

TTE: Transthoracic echocardiography
TEE: Transesophageal echocardiography
