# Supplementary material for: The effect of simulation of sectional human anatomy using ultrasound on students’ learning outcomes and satisfaction in echocardiography education: a pilot randomized controlled trial
Source: BMC Med Educ. 2024 May 3;24:494. doi: 10.1186/s12909-024-05337-x (PMC11069238; doi:10.1186/s12909-024-05337-x)
Supplement: Supplementary file 1 — Supplementary Material 1 [file 12909_2024_5337_MOESM1_ESM.docx]

**Supplementary material 1** The composition of the question bank

| **Question types** | Section content | Section position | Section function | Other types | Total |
| --- | --- | --- | --- | --- | --- |
| **Number** | 22 | 9 | 4 | 5 | 40 |

Note: 1. The section content includes: structure identification and cardiac cycle judgment

2. The section position includes: the position and orientation of the probe

3. The section function includes: the clinical uses

4. The other types involve multiple aspects above at the same time or involve other properties of the section
